# Telocytes in female reproductive system (human and animal)

**DOI:** 10.1111/jcmm.12843

**Published:** 2016-04-05

**Authors:** Veronika Aleksandrovych, Jerzy A. Walocha, Krzysztof Gil

**Affiliations:** ^1^ Department of PathophysiologyJagiellonian University, Medical CollegeKrakowPoland; ^2^ Department of AnatomyJagiellonian University, Medical CollegeKrakowPoland

**Keywords:** telocytes, telopodes, myometrium, uterus, Fallopian tube, sex steroid receptors, human placenta, CD34

## Abstract

Telocytes (TCs) are a newly discovered type of cell with numerous functions. They have been found in a large variety of organs: heart (endo‐, myo‐, epi‐ and pericardium, myocardial sleeves, heart valves); digestive tract and annex glands (oesophagus, stomach, duodenum, jejunum, liver, gallbladder, salivary gland, exocrine pancreas); respiratory system (trachea and lungs); urinary system (kidney, renal pelvis, ureters, bladder, urethra); female reproductive system (uterus, Fallopian tube, placenta, mammary gland); vasculature (blood vessels, thoracic duct); serous membranes (mesentery and pleura); and other organs (skeletal muscle, meninges and choroid plexus, neuromuscular spindles, fascia lata, skin, eye, prostate, bone marrow). Likewise, TCs are widely distributed in vertebrates (fish, reptiles, birds, mammals, including human). This review summarizes particular features of TCs in the female reproductive system, emphasizing their involvement in physiological and pathophysiological processes.

## History

Telocytes (TCs) were discovered in 2005 when L.M. Popescu's group from Bucharest, Romania, described a new type of cell that resides in the stroma of several organs, which became known as interstitial Cajal‐like cells (ICLC). This group named these cells as ICLC because of their apparent similarity with the canonical gastrointestinal cells of Cajal (ICC), the gut pacemaker cells. A few years later, in 2008, M.S. Faussone‐Pellegrini and her team from Florence, Italy, described ICLC in the muscle coat of the human gut and noticed they consistently differed from the ICC in both ultrastructure and immunophenotype. In 2010, after confirming the presence of this particular cell type in the stroma of many organs and characterized it by immunohistochemistry and electron microscopy, the two groups agreed they were describing a ‘novel’ cell type and that the name ICLC should be changed to a more appropriate one. From then on, this novel cell type became known as the TCs (using the Greek affix ‘Telos’) 1, 2.

## Characteristics of telocytes (morphology, contacts)

Telocytes have been shown to differ from fibroblasts and mesenchymal stem cells in terms of gene profile and proteomics, and in microRNA expression. They have the principal morphological characteristics contrasted with other types of cells 3. Currently, the most demonstrative and widespread methods for identification of TCs include transmission electron microscopy (TEM), immunohistochemistry and immunofluorescence. Transmission electron microscopy is considered the most accurate method for identifying TCs 4.

The TC has a small, oval‐shaped cellular body, containing a nucleus, surrounded by a small amount of cytoplasm. The cellular body average dimensions are, as measured on TEM images, 9.39 μm ± 3.26 μm (min = 6.31 μm; max = 16.42 μm). The nucleus occupies about 25% of the cell volume and contains clusters of heterochromatin attached to the nuclear envelope.

The perinuclear cytoplasm is rich in mitochondria (which occupy about 5% of the cell body) particularly in podoms, which contain a small Golgi complex, as well as the elements of rough and smooth endoplasmic reticulum and cytoskeletal elements (thin and intermediate filaments) 5, 6, 7.

The cell periphery is represented by the usual plasmalema, with no (or thin and discontinuous) basal lamina, and some caveolae (about 2–3% of cytoplasmatic volume; ~0.5 caveolae/μm of cell membrane length).

The shape of the TCs depends on the number of their telopodes Tps: piriform for one prolongation, spindle for two Tps, triangular for three, stellate, *etc*. Their spatial appearance is that of a polyhedron with a different number of vertices, depending on their TP number 1, 2, 7.

Telocytes have a variable number of Tps (very long cellular extensions), which are probably the longest cellular prolongations in the human body. Tps are made by an alternation of dilated portions, named podoms (250–300 nm), containing mitochondria and endoplasmic reticulum and podomers (~80 nm) with thin segments. The main characteristics of Tps:

1. Number: 1–5, frequently only 2–3 Tps are observed on a single section, depending on site and angle of section, since their 3D convolutions impede their observation in a very thin 2D section along the entire length of the TP their full length.
2. Length: from tens to hundreds of μm, as measured on EM images. However, under favourable condition in cell cultures, their entire length can be captured.
3. Thickness: an uneven caliber, mostly below 0.2 μm (resolving power of light microscopy), visible under electron microscopy, only 0.10 μm ± 0.05 μm (min = 0.03 μm; max = 0.24 μm)
4. Moniliform aspect, with many dilations along their length;
5. Presence of ‘Ca^2+^‐release units’ at the level of the dilations, accommodating mitochondria, elements of endoplasmic reticulum and caveolae;
6. Branching, with a dichotomous pattern;
7. Organization in a network—forming a labyrinthine system by tridimensional convolution and overlapping, communicating through gap junctions.

These characteristic features make Tps clearly different from neuronal dendrites, the processes of antigen‐presenting dendritic cells or fibroblasts and myofibroblasts 2, 8.

In uterine tissue, TCs can influence contractile activity of smooth muscle cells. They differed with pregnant states in telopodal width and podomic thickness, which can be considered to be related to their function 9, 10. Current studies showed the podomers are thicker in nonpregnant myometrium than in pregnant (~82 *versus* 75 nm), and the podoms were thicker in pregnant myometrium (~316 *versus* 269 nm) 4, 6.

Popescu and Enciu experimentally demonstrated that TCs morphology changes with ageing and by modifying the redox balance of the cell culture environment. Oxidative stress impairs the ability of TCs to form Tps and lessens the migration pathway length. Ageing further aggravated this effect 11.

Furthermore, TCs demonstrate specific direct (homocellular and heterocellular junctions) and/or indirect (chemical, paracrine/juxtacrine signalling, microvesicles and exosomes, sex hormone and microRNAs) contacts with various surrounding cells 1, 7, 8.

Telopodes are connected to each other by homocellular junctions and appear to form a 3D network in the interstitial space at the border of smooth muscle cell bundles. They also contain cytoskeleton elements. Connections between TCs‐exosomes‐intercellular junctions‐cytoskeleton form the equivalent of a primitive nervous system 9, 10, 12, 13.

Heterocellular nanocontacts^1^ were frequently described between TCs and myocytes, or TCs and immune cells. Their contacts with mast cells provide a reason to predict their participation in immunoreactions (mastocytes‐mediated immunoregulation/and immunosurveillance). Moreover, various studies have revealed that this class of cells play two roles in the immune response. During the physical process, TCs can be activated to maintain homeostasis to induce proliferation, differentiation and tissue regeneration. On the other hand, they initiate tissue inflammation to induce pathogenesis under some challenges 14.

Telocytes also surrounded stem cell niches with Tps and heterocellular contacts. In addition, they establish physical contacts with nerve endings, blood vessels and different types of progenitor cells. Accumulating studies have shown that TCs play an indisputable role in neo‐angiogenesis. As TCs have cytoskeleton elements (myosin‐14, periplakin), this predicts that they could be responsible for detecting smooth muscle cell stretch during the enlargement of the uterus in pregnancy 6, 13, 15, 16.

Telocytes release at least three types of extracellular vesicles: exosomes (45 ± 8 nm), ectosomes (128 ± 28 nm) and multivesicular cargos (1 ± 0.4 μm) from their Tps and, occasionally, from the cell body 4. Specifically, extracellular vesicles and/or exosomes are shed or released from Tps in uterine TCs. Mediators, such as interleukin (IL)‐6, VEGF and nitric oxide, are secreted from TCs. Growth factors, including IL‐6, VEGF, macrophage inflammatory protein 1α, macrophage inflammatory protein‐2 and monocyte chemoattractant protein 1, are significantly expressed along with additional cytokines, including IL‐2, IL‐10, IL‐13 and chemokines, such as growth‐related oncogene/keratinocyte‐derived chemokine in the secretome of cultured rodent cardiac TCs 17. Besides, in a renal ischaemia‐inflammatory injury model, TCs activated the nuclear factor kappa B signalling pathway and upregulated the mRNA levels of pro‐inflammatory cytokines such as IL‐1 and tumour necrosis factor‐α 17, 18.

## Gene expression profile of telocytes

Nowadays, increasing knowledge in the chromosomal gene expression profile of TCs can be a further step in research. Several studies have shown interconnections between TCs and genes of chromosomes 1, 2, 3, 4, 17 and 18. Three genes, Capn2, Fhl2 and Qsox1, were overexpressed in pulmonary TCs compared with the other cells. TCs enriched with Capn2 and Fhl2 may be associated with the regulation of tissue inflammation, injury, repair, immune responses or cell movement. Besides, as Capn2 is important for embryogenesis, it explains the involvement of TCs in morphogenesis and tissue homeostasis. The lower expression of chromosome 1 genes in TCs, as compared with other cells, was found for Ifi203 (interferon‐activated gene 203) and Tcea1 (transcription elongation factor A SII‐1) 11, 19.

In chromosome 2, 26 genes were overexpressed in TCs. One gene Myl9 (myosin, light polypeptide 9) was overexpressed most in TCs, different from other cells. Myl9 regulatory gene encodes the regulatory light chains of myosin II molecule, known to play a central role in cell adhesion, migration and division. There were six genes, *e.g*. Pltp, Gzf1, Polr1b, Tasp1, Zbtb34 and Zfp120, down‐expressed most in TCs, differentially from other cells. In chromosome 3, there were 13 up‐ and 59 down‐regulated genes in TCs 20.

Approximately 15% and 12% genes in chromosomes 17 and 18 were identified as TCs‐specific genes. Sixteen and 10 of TCs‐specific genes were up‐regulated, and 68 and 22 were down‐regulated in chromosome 17 and 18, as compared with other cells. Most of the observed genes in both chromosomes were immune‐associated, which supported the role of TCs in immune surveillance and immune homeostasis 14.

In chromosome 4, 17 genes were up‐ and 56 down‐regulated. Among up‐regulated genes were Akap2, Gpr153, Sdc3, Tbc1d2, which encode proteins involved in cell signalling pathways and cytoskeleton organization. It seems that TCs could integrate signals and autoregulate its own fate, integrating autophagy with endocytic trafficking 21.

## Immunohistochemical profile of telocytes

As any type of cells, TCs have own immunohistochemical profile. Current studies demonstrate expressions or (co‐) expression of different markers in TCs, which at the same time not are peculiar to a given organ. TCs are immunohistochemically positive for CD34 (Table 1), CD117/c‐Kit, plated‐derived growth factor receptor alpha and beta (PDGFRα and‐β), VEGF, inducible nitric oxide synthase (iNOS), calveolin‐1, vimentin, connexin 43, oestrogen and progesterone receptors (PRs), CD44, desmin, nestin and cadherin‐11, according to the TC location. In addition, they are immunohistochemically negative for procollagen 1, CD31/PECAM‐1 (endothelial cells), α‐smooth muscle actin (α‐SMA) (myofibroblasts, pericytes, and vascular SMCs), CD11c (dendritic cells and macrophages), CD90/Thy‐1 (fibroblasts) and, sometimes, c‐ kit/CD117 (mast cells). For instance, Tps of TCs from non‐pregnant myometrium are intensively positive for vimentin, a cytoskeleton protein 6, 9, 22, 23, 24, 25, 26, 27. Telocytes are also negative for CD68 and other markers associated with immune functions (CD1a, CD62‐P), suggesting a clear difference between TCs and macrophages. However, hypothetically TCs, myocytes and leucocytes could work together in pregnancy maintenance or in the onset of labour 6.

**Table 1:** Panel of primary antibodies for detecting CD34 in telocytes

Type of tissue	Antibody	Clone	Name of company	Dilution	Time
Human myometrium	Monoclonal mouse CD34	8G12	BD Immunocytometry Systems, San Jose, CA, USA	1:10	4 hrs, RT
Human myometrium	Goat anti‐CD34		R&D, Minneapolis, MN, USA	1:20	2 hrs, RT
Human myometrium	Goat polyclonal CD34	sc‐7045	Santa Cruz Biotechnoogy, Inc., Heidelberg, Germany	1:50	1 hr, RT
Human placenta	Monoclonal mouse anti‐human CD34	QBEnd‐10	Cat. No. M7165; Dako, Glostrup, Denmark	1:25	1 hr, RT
Human placenta	Mouse CD34	563	Cat. No. 550760; BD Biosciences	1:50	1 hr, RT
Human myometrium	Monoclonal mouse anti‐human CD34	QBEnd‐10	Cat. No. M7165; Dako	1:100	30 min., RT
				1:50	1 hr, RT
Human bladder	Monoclonal mouse anti‐human CD34		Cat. No. M7165; Dako	1:50	Overnight, RT

RT: room temperature.

It is important to note that TCs might have subpopulations. The expression of c‐kit receptors differs between TCs populations (possible site dependant). For instance, rat uterus tissue contains different types of immune positive TCs: c‐kit (−)/vimentin (+), c‐kit (+)/vimentin (+), c‐kit (+)/CD34 (+). This range might be basis of region‐specific TCs roles 13.

In the chorial villi, some TCs are c‐kit‐positive; and some CD34 positive A of the CD34 positive TC express vimentin and calveolin‐1, and some of them also c‐kit. In cultured cells from the same placental villi, some TCs are double positive for c‐kit and iNOS and others for c‐kit and VEGF 26.

## Electrophysiology

Telocytes are also involved in the electrical modulation of excitable tissue, such as the smooth muscle of the gut and uterus. They can spontaneously initiate electrical activity and modulate of glandular and immune activity 28. Ion channels, such as T‐type calcium and small‐conductance calcium‐activated potassium channels, are present in TCs 7.

T‐type calcium channels are present in TCs from human myometrium, which, in pregnancy and labour, could participate in the generation of endogenous bioelectric signals responsible for the regulation of the surrounding cell behaviour. Surprisingly, this might be the missing link for describing the molecular mechanisms by which TCs are involved in mechanical stretching during uterine enlargement in pregnancy. The expression of α‐subunit of T‐type calcium channels in TCs is less intense in the case of non‐pregnant myometrium 22.

Steroid hormones and oxytocin might mediate the higher expression of T‐type calcium channels in TCs derived from pregnant myometrium. As TCs have steroid hormone receptors, this might lead to frequent and sustained contractions able to trigger birth 29.

There is the opposite situation in the expression of small‐conductance calcium‐activated potassium channels isoform 3 (SK3). This type of ion channel has been identified in the myometrium of several species including human, mice and rats, but with great interspecies variation in the pattern of expression and regulation 30. The SK3 channels have been detected more often in non‐pregnant myometrium, whiereas pregnant myometrium showed a lack of them. A similar situation with SK3 expression in vascular endothelium is found during pregnancy. These are also expressed in TCs and are down‐regulated during pregnancy when they reduce contractility 28. Telocytes may represent a novel mechanism controlling the excitability of the uterine musculature. As has been suggested by Cretoiu *et al*. and Allix *et al*., a possible mechanism could be *via* a tyrosine‐kinase independent signalling pathway 23, 31.

In the myometrium, TCs contain only outward potassium‐rectifier channels, but no inward currents have been evidence so far. There also is a lack of regular slow waves of depolarization in TCs from myometrium.

## Laser‐stimulation of telocytes

The low‐level laser stimulation (LLLS) of TCs produces a higher growth rate of lateral telopodal extension in pregnant myometrium primary cultures as compared to non‐pregnant ones. The angel of deviation is more accentuated in TCs from human pregnant myometrium than in TCs from non‐pregnant myometrium. Therefore, some implications are emerging for low‐level laser therapy in uterine regenerative medicine. Telopodes from the pregnant uterus are more prone to extend upon LLLS compared to those from the non‐pregnant uterus. The required number of stimulations is sometimes three times higher than in pregnant myometrium. In both preparations, Tps seem to accumulate a big part of their resources near the stimulation area. Finger‐like structures were probed with the laser beam. In some experiments, the TP appears to be is breaking off its old connection, maintaining only thin ‘anchors’ beyond the point of stimulation.

Twenty‐five percent of TCs from pregnant uterus present a local thickening of the TP upon LLLS. The local thickening phenomenon was directly correlated with a delayed telopodal response to stimulation 9.

Some experiments have been carried out on the action of imatinib (c‐kit receptor antagonist) on the spontaneous and oxytocin‐induced contractions in human non‐pregnant myometrium and the myometrium of pregnant rabbits. C‐kit inhibition led to a reduction in both the amplitude and frequency of myometrial contraction in a dose‐dependent manner. Results show that TCs might be players in the coordination of uterine activity in a kit‐independent manner. This may be under hormonal influences, since express oestrogen and PRs. Also TCs could be modulators of the electrical excitability of underlying contractions, but are not necessarily pacemakers 6, 23, 32.

## Characteristics of TCs in pregnant/non‐pregnant condition

Numerous experiments have shown that the amount of TCs in endometrium and myometrium in rats changes depending on its reproductive state. In pregnant uteri endometrial TCs increase, compared with non‐pregnant, in spite of a significant decline in the number of myometrial TCs. Postpartum uteri show the highest significant count of myometrial TCs and non‐significant difference in endometrial TC count, as compared with the adult non‐pregnant group. The lowest count of both types of TCs occurs in the immature rat uteri, whereas the highest amount of endometrial TCs was detected in the pregnant uteri and the highest amount of myometrial TCs in the postpartum uteri. Telocytes produce electric slow waves that trigger and coordinate smooth muscle contractions; the decrease in myometrial TCs numbers in the pregnant rat uterus may explain the prevention of preterm delivery (uterine contractility before term). The increase after delivery may be connected with the involution of the uterus accompanied by myometrial contractions 33.

In one further example of involving TCs in pregnancy is their increase during that condition. Hence, Hatta *et al*. has suggested that TCs are frequently present in tissues that have a low cell density and significant space between neighbouring cells. Moreover, TCs express connexin 43, a gap junction protein, which most likely has a vital role in decidual maturation, as its decrease is associated with recurrent pregnancy loss 4, 34.

In a literature study, we have estimated the average number of TCs in human uterus. Telocytes constituted about 7% of the total cell number in non‐pregnant myometrial cell culture and about 3% of the entire cell population in the myometrium of adult non‐pregnant humans 33. This was not accompanied by any difference in the size of TC's exo‐ and ectosomes in pregnant and non‐pregnant myometrium 4.

In addition, the number of CD117‐positive cells is reduced during pregnancy under normal condition. This prevents myogenic contractions and early delivery, as CD‐117‐positive cells within the myometrium are involved in myogenic contractile mechanisms during pregnancy 32.

A study on the role of murine TCs pregnancy‐induced liver growth demonstrated the potential involvements of TCs in hepatic proliferative adaptations to pregnancy. An increase in CD34/PDGFR‐α positive TCs in pregnant liver accompanied by high level of hepatocyte proliferation 35.

## Main features of TCs in endometrium and Fallopian tubes

In the human endometrium, the location of TCs is contoured to the shape of the adjacent epithelial architecture. They may support the structure of the gland and nearby stroma as a functional unit by forming a scaffold around them. In addition, they may take part in loosening the matrix or secreting factors during decidual formation 34.

Telocytes, which were observed from rat's endometrium, stained positive for connexin 43, a protein involved in gap junctions. Connexin 43 is involved in maturation of decidua and its decreasing connection with recurrent pregnancy loss. Endometrial TCs may play the role of gap junctions and have impact on normal developing of pregnancy 33.

In the human Fallopian tube, TCs are located mainly in mucosa and muscular layer among smooth muscle fibres 36. Oviduct TCs numbers decline in women with endometriosis, tubal ectopic pregnancy and acute salpingitis. Probably, this can be caused by the overproduction of iNOS, COX‐2, LPO and estradiol in oviduct tissue, and TCs damage results. In acute salpingitis‐affected oviduct tissues, TCs levels were greatly decreased or even completely lost. They are severely damaged/degenerated with multiple ultrastructural abnormalities, such as loss of organelles, numerous swollen nuclei, mitochondrial and rough endoplasmic reticulum dilatation, cytoplasmic vacuolization, discontinuation or dissolution of Tps and swollen or loss of intercellular junctions as Tps and activated immunocyte (eosinophiles) with dense secretory granules respectively 37.

Damage and loss of TCs can change the activity of TCs‐stem cells and decrease tissue reparation or renewal capacity, subsequently inducing the development of tissue fibrosis within endometriosis‐affected oviduct. This will then cause Fallopian tube dysfunction and infertility. On the other hand, the reconstruction TCs network might be of great value for structural and functional reparation of fibrous disease of Fallopian tube 13, 25.

In addition, oviduct TCs express oestrogen receptor alpha and PR‐A, and thus might act as ‘hormonal sensors’, and their function may be in part under hormonal control. These receptors are specific for TCs localization. For example, TCs of gallbladder are negative for both types of receptors and gallbladder muscular contractions are not regulated by sex steroids. TCs were more often tested with anti PR‐A antibody. The PR‐A expression is recognized by most anti‐PR antibodies, whereas the PR‐B expression was not detected. It is possible that sex steroid levels could impact on TCs *via* connexin43‐mediated mechanism 38.

## TCs in human placenta

The placenta also contains functional TCs. They were discovered in the large stem villi, with their long, slender process surrounding the blood vessel wall, or interposed between arterioles and the trophoblast basement membrane in small stem villi. The TCs were connected by gap junctions in a network extending into the placental stroma between concentric layers formed by smooth muscle cells and myofibroblasts around large blood vessels. The TCs had close contacts with mast cells, Hofbauer cells,^2^ myofibroblasts and smooth muscle cells. Moreover, they have close contacts with different perivascular smooth muscle cells through different branches 26, 39.

In addition, TCs in human term placenta are positive for c‐kit, CD34, vimentin, caveolin‐1, VEGF and iNOS. Presumptive roles of TCs in term placenta are partly related to their immunologic profile. Firstly, as TCs are located near vessels and, as the placenta is a non‐innervated organ, they might be involved in blood flow regulation. Secondly, TCs express VEGF and might have impact on tissue remodelling. Finally, TCs appear frequently cited as an ‘intercellular bridge’ between two HBCs. They might play the role of immune surveillance. Moreover, TCs express steroid hormone receptors. This could have an impact on the development of connections between the placenta and uterus 26, 40.

Telocytes are identified close to small vessels and express angiogenesis‐related factors (VEGF, nitric oxide) and pro‐angiogenic microRNAs (miR126, miR130a, let‐7‐family, miR‐10, miR‐155 and miR‐503). Moreover, it has been shown that CD34 positive cells secrete vesicles (exosomes) that have an independent angiogenic activity, both *in vitro* and *in vivo* 41, 42. Likewise, TCs have been shown to express significant amounts of miR‐21, ‐22, ‐29, and ‐199a and oestrogen and PRs. Micro‐RNA can be involved in the pathogenesis of uterine leiomyomata, as had been proved by current genetic research. Consequently, TCs may have a significant impact on uterine leiomyoma formation 4, 43, 44.

## Possible role of TCs in female reproductive system

Numerous studies have been carried out in order to detect the properties and functions of TCs in female reproductive organs over the past 10 years. Apparently, these new cells play significant roles in normal physiological processes (and their pathologies) like myometrial contractility, immune responses, the development and secretions of the placenta, regulation of adaptive response to overcome vascular resistance during normal pregnancy.

The list also includes the role of TCs on angiogenesis and tissue development, which can be crucial in cancerogenesis, uterine leiomyoma formation, ectopic tubal pregnancy, endometriosis and salpingitis.

Telocytes are located in the neuromuscular spindles and participate in the control of muscle tone and motor activity. Telocytes form homo‐ and heterocellular contacts with adjacent cells and might even be able to control and regulate their activity, participating in tissue remodelling/renewal 6. An immune surveillance role was suggested for the network of TCs, located in human Fallopian tube 1. Tubal TCs were reported to be involved in the early onset of endometriosis and acute salpingitis 37. Moreover, the decrease in TCS caused dysregulation of oviduct motility, suggesting that tubal TCs impairment leads to the infertility of the tubal origin and even tubal ectopic pregnancy 13.

Telocytes could serve as the ‘hormonal sensors’ in human uterus, since there is an evidence of some uterine stromal cells that play a role in endometrial growth and differentiation in a hormone‐dependent manner 6. They also contribute to cell migration and proliferation of myometrial tissue 7. Thus, TCs may also be involved in the hyperplasia of endometrium.

Telocytes, myocytes and leucocytes could work together in pregnancy myometrium or in the onset of labour 6. They even might play a role in preventing premature uterine contractility 7.

Genetic and immunologic profiles of TCs give us an opportunity to explore its cooperation with immune cells. Applications in regenerative medicine may extend the therapeutic role of TCs in pathological conditions. We are only at the beginning of our explorations of the powers and the versatility of TCs.

## Conflicts of interest

The authors confirm that there are no conflicts of interest.

## Notes

1 Nanocontacts—heterocellular communications between TCs and cardiomyocytes, which do not belong to classic types of junctions found in textbooks. It has no typical features for different types of junctions known, except small nanopillars (10–15 nm/20–30 nm) that connect the cellular membranes. The bridging structure suggests a molecular interaction between the Tps and cardiomyocytes but their electron density is below the density of membranes, and isosurface reconstruction is not appropriate to imagine these nanostructures. Different sections through the reconstructed volume showed clusters of nanocontacts with symmetrical distribution, fastening the connection between TCs and cardiomyocytes.

2 Hofbauer cells (HBCs) are placental macrophages that are present in the villus across gestation. Despite their identification more than 100 years ago, their specific role in placental function remains largely unelucidated.

## References

[1] Faussone‐Pellegrini MS , Popescu LM . Telocytes. Biomol Concepts. 2011; 2: 481–9. 25962049 10.1515/BMC.2011.039

[2] Popescu LM , Faussone‐Pellegrini MS . TELOCYTES – a case of serendipity: the winding way from interstitial cells of Cajal (ICC), *via* interstitial Cajal‐like cells (ICLC) to TELOCYTES. J Cell Mol Med. 2010; 14: 729–40. 20367664 10.1111/j.1582-4934.2010.01059.x PMC3823108

[3] Niculite CM , Regalia TM , Gherghiceanu M , *et al* Dynamics of telopodes (telocytes prolongations) in cell culture depends on extracellular matrix protein. Mol Cell Biochem. 2015; 398: 157–64. 25240414 10.1007/s11010-014-2215-z PMC4229650

[4] Roatesi I , Radu BM , Cretoiu D , *et al* Uterine telocytes: a review of current knowledge. Biol Reprod. 2015; 93: 10. 25695721 10.1095/biolreprod.114.125906

[5] Bei Y , Wang F , Yang C , *et al* Telocytes in regenerative medicine. J Cell Mol Med. 2015; 19: 1441–54. 26059693 10.1111/jcmm.12594 PMC4511344

[6] Cretoiu SM , Cretoiu D , Marin A , *et al* Telocytes: ultrastructural, immunohistochemical and electrophysiological characteristics in human myometrium. Reproduction. 2013; 145: 357–70. 23404846 10.1530/REP-12-0369 PMC3636525

[7] Cretoiu SM , Popescu LM . Telocytes revisited. Biomol Concepts. 2014; 5: 353–69. 25367617 10.1515/bmc-2014-0029

[8] Dawidowicz J , Szotek S , Matysiak N , *et al* Electron microscopy of human fascia lata: focus on telocytes. J Cell Mol Med. 2015; 19: 2500–6. 26311620 10.1111/jcmm.12665 PMC4594691

[9] Campeanu RA , Radu BM , Cretoiu SM , *et al* Near‐infrared low‐level laser stimulation of telocytes from human myometrium. Lasers Med Sci. 2014; 29: 1867–74. 24870411 10.1007/s10103-014-1589-1 PMC4215113

[10] Cretoiu SM , Cretoiu D , Popescu LM . Human myometrium – the ultrastructural 3D network of telocytes. J Cell Mol Med. 2012; 16: 2844–9. 23009098 10.1111/j.1582-4934.2012.01651.x PMC4118253

[11] Enciu AM , Popescu LM . Telopodes of telocytes are influenced *in vitro* by redox conditions and ageing. Mol Cell Biochem. 2015; 410: 165–74. 26335900 10.1007/s11010-015-2548-2

[12] Smythies J , Edelstein L . Telocytes, exosomes, gap junctions and the cytoskeleton: the makings of a primitive nervous system? Front Cell Neurosci. 2013; 7: 278. 24427115 10.3389/fncel.2013.00278 PMC3879459

[13] Yang XJ , Yang J , Liu Z , *et al* Telocytes damage in endometriosis‐affected rat oviduct and potential impact on fertility. J Cell Mol Med. 2015; 19: 1720–8. 25388530 10.1111/jcmm.12427 PMC4407595

[14] Wang J , Ye L , Jin M , *et al* Global analyses of Chromosome 17 and 18 genes of lung telocytes compared with mesenchymal stem cells, fibroblasts, alveolar type II cells, airway epithelial cells, and lymphocytes. Biol Direct. 2015; 10: 9. 25888380 10.1186/s13062-015-0042-0 PMC4355521

[15] Ciontea SM , Radu E , Regalia T , *et al* C‐kit immunopositive interstitial cells (Cajal‐ type) in human myometrium. J Cell Mol Med. 2015; 19: 407–1720. 10.1111/j.1582-4934.2005.tb00366.x PMC6740058 15963260

[16] Gherghiceanu M , Popescu LM . Heterocellular communication in the heart: electron tomography of telocyte‐myocyte junctions. J Cell Mol Med. 2011; 15: 1005–11. 21426485 10.1111/j.1582-4934.2011.01299.x PMC3922684

[17] Chi C , Jiang XJ , Su L , *et al* *In vitro* morphology, viability and cytokine secretion of uterine telocyte‐activated mouse peritoneal macrophages. J Cell Mol Med. 2015; 19: 2741–50. 26471943 10.1111/jcmm.12711 PMC4687714

[18] Li L , Lin M , Li L , *et al* Renal telocytes contribute to the repair of ischemically injured renal tubules. J Cell Mol Med. 2014; 18: 1144–56. 24758589 10.1111/jcmm.12274 PMC4508154

[19] Sun X , Zheng M , Zhang M , *et al* Differences in the expression of chromosome 1 genes between lung telocytes and other cells: mesenchymal stem cells, fibroblasts, alveolar type II cells, airway epithelial cells and lymphocytes. J Cell Mol Med. 2014; 18: 801–10. 24826900 10.1111/jcmm.12302 PMC4119386

[20] Zheng M , Sun X , Zhang M , *et al* Variations of chromosomes 2 and 3 gene expression profiles among pulmonary telocytes, pneumocytes, airway cells, mesenchymal stem cells and lymphocytes. J Cell Mol Med. 2014; 18: 2044–60. 25278030 10.1111/jcmm.12429 PMC4244019

[21] Song D , Cretoiu D , Zheng M , *et al* Comparison of Chromosome 4 gene expression profile between lung telocytes and other local cell types. J Cell Mol Med. 2016; 20: 71–80. 26678350 10.1111/jcmm.12746 PMC4717865

[22] Cretoiu SM , Radu BM , Banciu A , *et al* Isolated human uterine telocytes: immunocytochemistry and electrophysiology of T‐type calcium channels. Histochem Cell Biol. 2015; 143: 83–94. 25212658 10.1007/s00418-014-1268-0 PMC4286651

[23] Cretoiu SM , Simionescu AA , Caravia L , *et al* Complex effects of imatinib on spontaneous and oxytocin‐induced contractions in human non‐pregnant myometrium. Acta Physiol Hung. 2011; 98: 329–38. 21893472 10.1556/APhysiol.98.2011.3.10

[24] Duquette R , Shmygol A , Vaillant C , *et al* Vimentin‐positive, c‐kit negative interstitial cells in human and rat uterus: a role in pacemaking? Biol Reprod. 2005; 72: 276–83. 15385413 10.1095/biolreprod.104.033506

[25] Popescu LM , Ciontea SM , Cretoiu D . Interstitial Cajal‐like cells in human uterus and fallopian tube. Ann N Y Acad Sci. 2007; 1101: 139–65. 17360808 10.1196/annals.1389.022

[26] Suciu L , Popescu LM , Gherghiceanu M , *et al* Telocytes in human term placenta: morphology and phenotype. Cells Tissues Organs. 2010; 192: 325–39. 20664249 10.1159/000319467

[27] Vannucchi MG , Traini C , Guasti D , *et al* Telocytes subtypes in human urinary bladder. J Cell Mol Med. 2014; 18: 2000–8. 25139461 10.1111/jcmm.12375 PMC4244015

[28] Edelstein L , Smythies J . The role of telocytes in morphogenetic bioelectrical signaling: once more unto the breach. Front Mol Neurosci. 2014; 7: 41. 24860423 10.3389/fnmol.2014.00041 PMC4026729

[29] Hutchings G , Williams O , Cretoiu D , *et al* Myometrial interstitial cells and the coordination of myometrial contractility. J Cell Mol Med. 2009; 13: 4268–82. 19732238 10.1111/j.1582-4934.2009.00894.x PMC4496132

[30] Ranbek M , Nazemi S , Ødum L , *et al* Expression of the small conductance Ca^2+^‐ activated potassium cannel subtype 3 (SK3) in rat uterus after stimulation with 17β‐estradiol. PLoS ONE. 2014; 9: e87652. 24505302 10.1371/journal.pone.0087652 PMC3914860

[31] Allix S , Reyes‐Gomez E , Aubin‐Houzelstein G , Noël D , Tiret L , Panthier JJ , Bernex F . Uterine contractions depend on KIT‐positive interstitial cells in the mouse: genetic and pharmacological evidence. Biol Reprod. 2008; 79: 510‐7. 18480468 10.1095/biolreprod.107.066373

[32] Horn LC , Meinel A , Hentscel B . c‐kit/CD 117 positive cells in the myometrium of pregnant women and those with uterine endometriosis. Arch Gynecol Obstet. 2012; 286: 105–7. 22271238 10.1007/s00404-012-2220-y

[33] Salama NM . Immunohistochemical characterization of telocytes in rat uterus in different reproductive stages. Egyptian J Histol. 2013; 36: 185–94.

[34] Hatta K , Huang ML , Weisel RD , *et al* Culture of rat endometrial telocytes. J Cell Mol Med. 2012; 16: 1392–6. 22551155 10.1111/j.1582-4934.2012.01583.x PMC3823209

[35] Wang F , Bei Y , Zhao Y , *et al* Telocytes in pregnancy‐induced physiological liver growth. Cell Physiol Biochem. 2015; 36: 250–8. 25967964 10.1159/000374068

[36] Popescu LM , Ciontea SM , Cretoiu D , *et al* Novel type of interstitial cell (Cajal‐like) in human fallopian tube. J Cell Mol Med. 2005; 9: 479–523. 15963270 10.1111/j.1582-4934.2005.tb00376.x PMC6740321

[37] Yang J , Chi C , Liu Z , *et al* Ultrastructure damage of oviduct telocytes in rat model of acute salpingitis. J Cell Mol Med. 2015; 19: 1720–8. 25753567 10.1111/jcmm.12548 PMC4511368

[38] Cretoiu SM , Cretoiu D , Suciu L , *et al* Interstitial Cajal‐like cells of human Fallopian tube express estrogen and progesterone receptors. J Mol Histol. 2009; 40: 387–94. 20063045 10.1007/s10735-009-9252-z

[39] Suciu L , Popescu LM , Gherghiceanu M . Human placenta: *de visu* demonstration of interstitial Cajal‐like cells. J Cell Mol Med. 2007; 11: 590–7. 17635651 10.1111/j.1582-4934.2007.00058.x PMC3922366

[40] Zheng Y , Bai C , Wang X . Telocyte morphologies and potential roles in diseases. J Cell Physiol. 2012; 227: 2311–7. 21928344 10.1002/jcp.23022

[41] Manole CG , Cismasiu V , Gherghiceanu M , *et al* Experimental acute myocardial infarction: telocytes involvement in neo‐angiogenesis. J Cell Mol Med. 2011; 15: 2284–96. 21895968 10.1111/j.1582-4934.2011.01449.x PMC3822940

[42] Ullah S , Yang P , Zhang Q , *et al* Identification and characterization of telocytes in the uterus of the oviduct in the Chinese soft shelled turtle, *Pelodiscus sinensis*: TEM evidence. J Cell Mol Med. 2014; 18: 2385–92. 25230849 10.1111/jcmm.12392 PMC4302644

[43] Cismasiu VB , Radu E , Popescu LM . miR‐193 expression differentiates telocytes from other stromal cells. J Cell Mol Med. 2011; 15: 1071–4. 21447044 10.1111/j.1582-4934.2011.01325.x PMC3822620

[44] Zavadil J , Ye H , Liu Z , *et al* Profiling and functional analyses of microRNAs and their target gene products in human uterine leiomyomas. PLoS ONE. 2010; 5: e12362. 20808773 10.1371/journal.pone.0012362 PMC2927438

